# New Approaches in Cold Storage of Fruits: Impact of Postharvest Spermidine and Salicylic Acid Applications on Phenolic Compounds and Quality Characteristics of Raspberry Fruits

**DOI:** 10.1002/fsn3.70389

**Published:** 2025-06-05

**Authors:** Melekşen Akın, Sadiye Peral Eyduran, Kenan Çelik, Akgül Taş, Muttalip Gundogdu

**Affiliations:** ^1^ Department of Horticulture, Faculty of Agriculture Iğdır University Iğdır Türkiye; ^2^ Department of Horticulture, Faculty of Agriculture Muğla Sıtkı Kocaman University Muğla Türkiye; ^3^ GAP International Agricultural Research and Training Center Diyarbakır Türkiye; ^4^ Department of Plant and Animal Production, Seben İzzet Baysal Vocational School Bolu Abant Izzet Baysal University Bolu Türkiye; ^5^ Department of Horticulture, Faculty of Agriculture Bolu Abant Izzet Baysal University Bolu Türkiye

**Keywords:** organic acids, phenolic compounds, quality characteristics, raspberry, storage, vitamin C

## Abstract

In this study, raspberry fruits were treated with spermidine, salicylic acid, and spermidine + salicylic acid before storage, and the fruits were stored for 5, 10, and 15 days. As a result of the study, increases in weight loss, pH, decay, and respiration rates, and decreases in soluble solid content (SSC) were determined in the control group fruits at different storage periods according to different spermidine and salicylic acid treatments. In terms of TA, significantly lower values (5 days, spermidine: 0.91%, spermidine + salicylic acid: 0.90%; 15 days, spermidine + salicylic acid: 0.68%) were found in fruits in which spermidine was used, and higher TA values (10 days, salicylic acid: 0.85%; 15 days, salicylic acid: 0.78%) were found in fruits in which salicylic acid was used. In terms of biochemical parameters, it was observed that phenolic compounds were more preserved in 1.0 mM spermidine + salicylic acid‐treated fruits, and organic acids were more preserved in both 1.0 mM spermidine and spermidine + salicylic acid‐treated fruits. Vitamin C was better preserved in all treated fruits compared to control fruits. In addition, the dominant organic acid in raspberry fruits was citric acid, followed by malic acid and vitamin C, respectively (citric acid, malic acid and vitamin C contents at the end of 15 days: 703.45 mg 100 g^−1^, 134.81 mg 100 g^−1^ and 16.13 mg 100 g^−1^, respectively). The most abundant phenolic compound in fruits was catechin, followed by rutin and chlorogenic acid (catechin, rutin and chlorogenic acid contents at the end of 15 days: 35.86 mg 100 g^−1^, 12.34 mg 100 g^−1^ and 4.58 mg 100 g^−1^, respectively). The study concluded that the exogenous application of 1.0 mM spermidine + salicylic acid can be used as a post‐harvest tool to maintain the quality characteristics and storage life of raspberry fruit.

## Graphical abstract

This study was carried out to determine the effects of postharvest spermidine and salicylic acid applications on changes in organic acid and quality characteristics of raspberry fruits during storage. It was determined that spermidine and salicylic acid applications prevented weight loss of fruits and preserved other quality characteristics during storage more than the control group. It was also observed that it prevented changes in phenolic compound and organic acid contents of fruits during storage more than the control group.

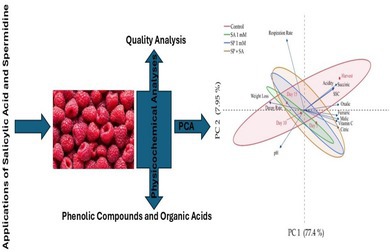

## 1 Introduction

Raspberry, which belongs to the genus *Rubus* spp. of the Rosaceae family, is a fruit belonging to the taxonomic species *Rubus idaeus* Linnaeus (Rosaceae: Rosales) (Hummer 2010). *R. idaeus* is native to the Americas and Europe, but can grow widely wherever temperate climatic conditions are observed. In Türkiye, this fruit is widely cultivated especially in the provinces of the Aegean coast, Black Sea, and Marmara regions (Erturk and Gecer 2012; TÜİK 2024).

Raspberry fruits are rich in antioxidants, anthocyanins, vitamin C, minerals, proteins, fatty acids, and carbohydrates (Nowak et al. 2018). It is known that these fruits are widely preferred by many dietitians and health professionals thanks to their many benefits, such as facilitating digestion, strengthening immunity, energizing the body, and regulating blood sugar (Li et al. 2019).

Although raspberry fruits can keep fresh‐like quality for certain periods of time, many adverse factors such as unfavorable climatic conditions during fruit cultivation, inadequate maintenance such as irrigation and fertilization, prolonged transportation conditions, and short storage time of the fruit can have direct negative effects on various quality attributes of the fruit (Hosseini et al. 2018). Among these, especially the limited storage time of a fruit is a major challenge in maintaining fruit freshness characteristics. Several studies have proposed various postharvest techniques to preserve fruit quality during storage. These include modified atmosphere storage, refrigeration, and ultraviolet (UV) treatments (Moradinezhad and Dorostkar 2021). Furthermore, the use of methyl jasmonate (Bagheri and Esna‐Ashari 2022), hot water treatments combined with calcium lactate (Naser et al. 2018), as well as hydrogen sulfide in conjunction with controlled atmosphere conditions, and the application of edible coatings (Sortino et al. 2022), have been reported to effectively reduce quality deterioration and maintain the biochemical integrity of fruits during storage. Therefore, methods to enhance fruit storage are needed. Indeed, in recent years, polyamine treatments such as spermidine have been widely used to prolong the storage period of fruits and maintain fruit quality characteristics (Orman et al. 2024).

Although polyamines can be found naturally in various plants, they can also be used externally to prolong fruit life. Spermidine, which is naturally present in all organs and cells of plants, plays an important role in fruit growth, development and physiology, especially in postharvest life. Thanks to its antioxidant properties, this compound also has stress‐neutralizing effects on plants (Liu et al. 2006). It is also known that quality parameters such as fruit weight, decay‐respiration rate, water soluble solids content and titratable acidity can be significantly improved by postharvest application of this compound in various fruits (Jalali et al. 2023). Additionally, the plant hormone ‘salicylic acid’, which naturally occurs in plants and is recognized for its ability to mitigate stress‐related effects, has also been utilized in recent years (Khalil et al. 2022). By applying salicylic acid to various fruits such as plums, apricots, sweet cherries and peaches, especially after harvest, the direct defense system in the fruit is activated and thus fruit quality characteristics can be maintained better and for longer periods (Valero et al. 2011; Mithöfer and Boland 2012; Tareen et al. 2012; Davarynejad et al. 2015; Asif et al. 2023).

This study aimed to investigate the effects of spermidine, salicylic acid, and spermidine + salicylic acid treatments on various physical and biochemical properties of raspberry fruit stored for different periods (5, 10, and 15 days). In the literature review, no study was found that examined the effects of spermidine and salicylic acid applications on the phenolic compound and organic acid contents of stored raspberry fruit. In this study, specific phenolic compounds and organic acids were examined, and new findings were presented to the literature in this field. It is important to examine the different doses of spermidine and salicylic acid and their interaction with other applications in more detail in the future in raspberry storage.

## 2 Materials and Methods

### 2.1 Materials

The raspberry garden was established with a row spacing of 2.5 m and an in‐row spacing of 1 m, and it was irrigated using the drip irrigation method. The soil pH was determined to be between 6.0 and 6.5 on average. Pruning operations were carried out, and weeds were removed from the garden. Raspberry fruits were harvested by hand when the SSC content was approximately 10%–11%. After harvest, the samples were placed in labeled containers, stored in ice boxes, and quickly transported to the laboratory for analysis. The experimental design consisted of three replications (1 kg fruit per replicate), 3 treatments [spermidine (1.0 mM), salicylic acid (1.0 mM) and spermidine + salicylic acid (1.0 mM)] and storage periods of 5, 10, and 15 days. Fruits were immersed in 0 mM (control, pure water), 1.0 mM spermidine, and salicylic acid solutions for 30 min. After treatment, the fruits were dried with blotting paper, placed in perforated polyethylene (PE) bags (30 cm × 40 cm, 0.01 mm thickness, 1 kg capacity) and stored under cold conditions (5°C ± 0.5°C and 90% ± 5% RH) for up to 30 days. Readings were taken at harvest (at the beginning of storage) for all treatments, including the control, and every 5 days during the storage period.

### 2.2 Determination of Weight Loss and Decay Rate

A digital precision balance with an accuracy of 0.01 g was used to assess the weight loss of raspberry fruits during storage. Weight loss was calculated as a percentage using the formula provided by Hosseini et al. (2018).

$$\begin{array}{c} (\text{Fruit weight}\;\mathrm{at}\;\text{the beginning of storage}-\text{Fruit weight}\\ \text{during storage})/\left(\text{Fruit weight during storage}\right)\times 100 \end{array}$$

Here, initial fruit weight refers to the fresh weight of raspberry fruits before storage, while fruit weight at the time of storage corresponds to the weight of the same raspberry fruits measured at a specific point during storage. This formula allows researchers to quantify the percentage reduction in fruit weight over time, reflecting moisture loss or other factors that contribute to weight changes.

The decay rate of raspberry fruits was assessed using a subjective scoring system to categorize the extent of decay observed on the fruit surface. The decay scoring scale was defined as follows: 0: No visible decay on the fruit. (1) Light rot, affecting no more than 25% of the surface of the fruit. (2) Moderate rot, affecting 25%–50% of the surface of the fruit. (3) Severe rot is when more than 50% of the surface of the fruit is affected. The method described by Cao et al. (2010) was applied to measure the overall decay rate of the fruit. The decay rate was calculated by the following formula:

$$\left(1\times \text{FN1}\;2\times \text{FN2}\;3\times \text{FN3}\right)\times 100/\left(3\times \mathrm{FN}\right)$$

Where FN is the total number of fruits, FN1, FN2, and FN3 are the number of fruits representing different decay scores.

### 2.3 Determination of Soluble Solids Content, Titratable Acidity, pH and Respiration Rate

Soluble solids content (SSC) was measured with a portable handheld refractometer (ATC, BX50, Türkiye). Titratable acidity (TA) was determined by titrating 10 mL of juice diluted with 10 mL of distilled water against 0.1 N NaOH using phenolphthalein as an indicator according to Hanif et al. (2020). In addition, pH was measured using a benchtop pH meter (Thermo, OrionStar A111, USA). To determine respiration rate, 100 g of fruit per replicate were placed in 2000 mL glass bottles with a CO_2_ sensor probe (Testo 535, Germany) inserted through a hole in the bottle cap. The probe and cap were sealed with parafilm to prevent CO_2_ escape (Huang et al. 2013). All experiments were conducted in a controlled environment, and respiration rate was recorded in mg CO_2_ kg^−1^ h^−1^. The data obtained were determined by the following formulas.

$$\mathrm{RR}=\frac{\Delta {\mathrm{CO}}_{2}\times M{\mathrm{CO}}_{2}\times V_{h}}{\mathrm{Vm}\times m\times \mathrm{\Delta t}}$$

$$\Delta {\mathrm{CO}}_{2}={\mathrm{CO}}_{2}\left(t_{2}\right)-{\mathrm{CO}}_{2}\left(t_{1}\right)$$

$$V_{m}=\frac{R\times T}{P}$$

where, RR: Respiration rate, mg CO_2_ kg‐1 h‐1; ΔCO_2_: CO_2_ volumetric concentration, ppm, 10–6 L L^−1^; MCO_2_: CO_2_ gas molecular weight, 44.01 g mol^−1^; *V*_h_: Bottle volume, L; *m*: Weight of single kernel wheat with shells, kg; Δ*t*: Trial period, h; CO_2_(t1): Initial CO_2_ concentration, ppm, 10–6 L L^−1^; CO_2_(t2): CO_2_ concentration at the end of the trial period, ppm, 10–6 L L^−1^; *V*_m_: Molar volume of gas, L mol^−1^; *R*: Gas constant, 0.08206 L^−1^ mol^−1^ 1 K^−1^; *T*: Temperature, K; *P*: Pressure, atm.

### 2.4 Determination of Organic Acids

Organic acids were identified using calibration curves prepared with standards (100–800 ppm, 99% purity, Sigma‐Aldrich, Taufkirchen, Germany). 250 g of raspberry portions were homogenized, and 50 g of the homogenate was diluted with distilled water (1:3). 25 mL of an extraction solvent of 0.009 N H_2_SO_4_ was added, and the mixture was shaken for 1 h. It was then centrifuged at 15,000 *g* for 15 min. The supernatant was filtered through rough paper and a 0.45 μm membrane (Millipore, Germany) and purified with a SEP‐PAK C18 cartridge. Analysis was performed using an Agilent 1100 HPLC system with a Bio‐Rad HPX‐87H column. 0.009 N H_2_SO_4_ detected at 254 and 280 nm was used as the mobile phase (Bevilacqua and Califano 1989). Results are expressed as mg/100 g FW according to calibration curves.

### 2.5 Determination of Vitamin C

Vitamin C in raspberry extracts (100 g) was extracted using 2.5% (wt/vol) metaphosphoric acid (Sigma, M6285, 33.5%) and centrifuged at 6500 rpm for 10 min at 4°C. Then, a 0.5 mL aliquot was diluted to 10 mL with the same acid solution. The supernatant was filtered through a 0.45 μm PTFE syringe filter (Phenomenex, UK) and analyzed on a C18 column (Phenomenex Luna C18, 250 × 4.60 mm, 5 μm) at 25°C. The mobile phase was acidified with double distilled water (pH 2.2, H_2_SO_4_) at a flow rate of 1 mL/min. Analysis was performed at 254 nm using a DAD detector. Quantification was based on L‐ascorbic acid standards (Sigma A5960) at 50, 100, 500, 1000, and 2000 ppm (Cemeroğlu 2007) and results were expressed as mg/100 g FW.

### 2.6 Determination of Phenolic Compounds

Phenolic compounds were analyzed using a modified HPLC method (Rodriguez‐Delgado et al. 2001). Standards for gallic acid, protocatechic acid (20–120 ppm), chlorogenic acid (200–1200 ppm), caffeic, vanillic, ferulic, and p‐coumaric acids (50–300 ppm) were prepared together with calibration curves (Sigma‐Aldrich, 99% purity, Taufkirchen, Germany). Flavonoid standards included rutin (50–300 ppm), quercetin (100–600 ppm) and catechin (500–2500 ppm). Fruit samples (100 g) were homogenized, shaken (Heidolph Unimax 1010, Germany) for 1 h, and diluted with water (1:1). The extract was centrifuged (15,000 rpm, 15 min), filtered (coarse paper and 0.45 μm membrane) and analyzed by HPLC (Agilent, USA) using a 250 × 4.6 mm, 4 μm ODS column (HiChrom, USA). The mobile phases consisted of methanol:acetic acid:water (10:2:28 and 90:2:8). Analysis was performed at 254 and 280 nm with a flow rate of 1 mL/min and an injection volume of 20 μL. Results are expressed as mg/100 g fresh weight.

### 2.7 Statistical Analysis

The characteristics of raspberry fruits were analyzed using two‐way ANOVA to assess the effects of two independent factors and their interaction. Post hoc comparisons were performed by Student's *t*‐test with significance set at *p* < 0.05. Statistical analyses were performed using JMP 13 software (SAS Institute Inc., Cary, NC, USA), which allows a detailed examination of main and interaction effects. Results included *F*‐values, *p*‐values, and treatment group means with standard errors to ensure clarity and transparency. This approach provided robust and reproducible results on factors affecting raspberry quality.

## 3 Results

### 3.1 Weight Loss, Soluble Solids Content, Titratable Acidity and pH

**FIGURE 1:**
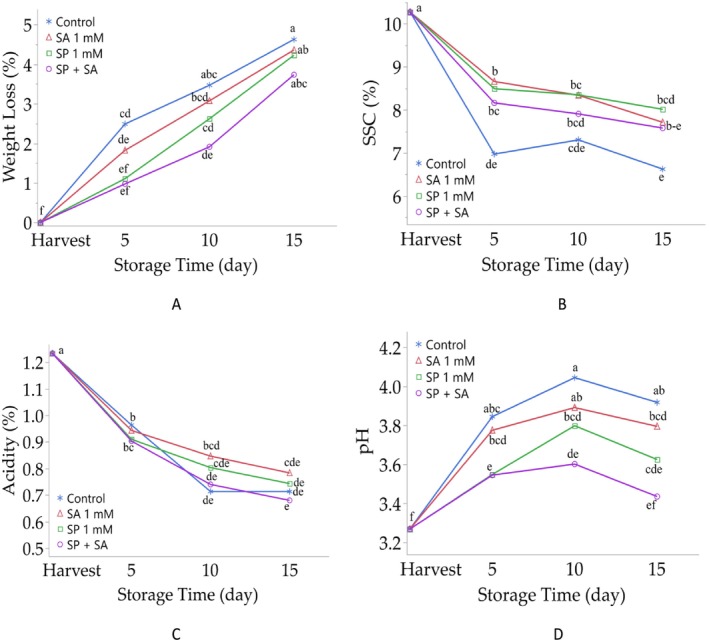
Effect of postharvest spermidine and salicylic acid application on weight loss (A), SSC: Soluble solid content (B), acidity (C), and pH (D) contents of raspberry fruits during storage. Different letters on top of the storage periods indicate significant differences at *p* ≤ 0.05.

The differences between the weight losses of control and treatments during storage of raspberry fruits were significant (*p* < 0.001). The weight losses increased with increasing storage time (5, 10 and 15 days) in control and all treatments. In this study, weight losses following all the applied treatments throughout the storage period [weight losses in different storage periods; 5 days: spermidine (1.11%), salicylic acid (1.82%) and spermidine + salicylic acid (0.97%); 10 days: spermidine (2.62%), salicylic acid (3.08%) and spermidine + salicylic acid (1.91%); 15 days: spermidine (4.23%), salicylic acid (4.36%) and spermidine + salicylic acid (3.74%)] showed significantly lower weight losses than the control group fruits (weight losses at different storage periods; 5 days: 2.49%, 10 days: 3.47%, 15 days: 4.63%). In addition, among the different treatments used in the study at all three storage periods, the least weight loss was observed in fruits treated with spermidine + salicylic acid (Table 1, Figure 1A).

**TABLE 1:** Impact of different doses of spermidine and salicylic acid applications on quality properties of raspberry fruits during storage.

Storage time (day)	Weight lost (%)	Decay rate (%)	SSC (%)	Acidity (%)	pH	Respiration rate (mg CO_2_ kg^−1^ h^−1^)
Harvest	0.00 ± 0.00d	0.00 ± 0.00d	10.27 ± 0.90a	1.23 ± 0.08a	3.27 ± 0.05b	6.21 ± 0.28a
5	1.60 ± 0.30c	2.91 ± 0.55c	8.07 ± 0.25b	0.93 ± 0.02b	3.68 ± 0.05a	4.41 ± 0.50b
10	2.77 ± 0.26b	5.04 ± 0.48b	7.98 ± 0.17b	0.78 ± 0.03c	3.83 ± 0.06a	6.20 ± 0.33a
15	4.24 ± 0.19a	7.72 ± 0.35a	7.48 ± 0.19b	0.73 ± 0.02c	3.69 ± 0.06a	6.41 ± 0.20a
SP and SA × Storage time interaction						
Harvest	0.00 ± 0.00f	0.00 ± 0.00f	10.27 ± 0.90a	1.23 ± 0.08a	3.27 ± 0.05f	6.21 ± 0.28bcd
Day 5						
Control	2.49 ± 0.89 cd	4.54 ± 1.62 cd	6.97 ± 0.53de	0.96 ± 0.05b	3.84 ± 0.04abc	7.10 ± 0.15ab
SP 1 mM	1.11 ± 0.05ef	2.02 ± 0.09ef	8.49 ± 0.27b	0.91 ± 0.02bc	3.55 ± 0.10e	3.56 ± 0.39f
SA 1 mM	1.82 ± 0.69de	3.32 ± 1.26de	8.66 ± 0.35b	0.94 ± 0.04b	3.77 ± 0.06bcd	3.70 ± 0.56f
SP + SA (1 mM)	0.97 ± 0.09ef	1.77 ± 0.16ef	8.16 ± 0.27bc	0.90 ± 0.02bc	3.54 ± 0.06e	3.27 ± 0.27f
Day 10						
Control	3.47 ± 0.67abc	6.32 ± 1.23abc	7.30 ± 0.14 cde	0.71 ± 0.05de	4.04 ± 0.10a	6.30 ± 0.23bcd
SP 1 mM	2.62 ± 0.58 cd	4.78 ± 1.05 cd	8.35 ± 0.34bc	0.80 ± 0.06cde	3.80 ± 0.04bcd	5.05 ± 0.41e
SA 1 mM	3.08 ± 0.13bcd	5.60 ± 0.24bcd	8.34 ± 0.12bc	0.85 ± 0.04bcd	3.89 ± 0.13ab	5.87 ± 0.26de
SP + SA (1 mM)	1.91 ± 0.16de	3.48 ± 0.29de	7.91 ± 0.40bcd	0.74 ± 0.03de	3.60 ± 0.07de	7.60 ± 0.64a
Day 15						
Control	4.63 ± 0.18a	8.43 ± 0.33a	6.62 ± 0.27e	0.71 ± 0.04de	3.92 ± 0.03ab	7.05 ± 0.55abc
SP 1 mM	4.23 ± 0.35ab	7.70 ± 0.63ab	8.01 ± 0.34bcd	0.74 ± 0.06de	3.62 ± 0.12cde	6.50 ± 0.28bcd
SA 1 mM	4.36 ± 0.24ab	7.94 ± 0.43ab	7.71 ± 0.16b‐e	0.78 ± 0.06cde	3.79 ± 0.04bcd	6.13 ± 0.25b‐e
SP + SA (1 mM)	3.74 ± 0.62abc	6.80 ± 1.13abc	7.58 ± 0.24b‐e	0.68 ± 0.03e	3.43 ± 0.08ef	5.95 ± 0.31cde
ANOVA						
*F* storage time	29.11***	29.16***	9.82***	38.12***	6.64**	6.59**
*F* storage time × SP and SA	9.93***	9.95***	5.47***	10.14***	8.18***	13.76***

*Note:* Different letters in the same column indicate statistical differences at *p* ≤ 0.05. *, **, *** indicate *p* ≤ 0.05, 0.01, and 0.001, respectively.

During the storage of raspberry fruits, the differences between different treatments and the control group were found to be significant (*p* < 0.001). In this study, all the applied treatments throughout the storage period [at different storage periods; 5 days: spermidine (8.49%), salicylic acid (8.66%) and spermidine + salicylic acid (8.16%); 10 days: spermidine (8.35%), salicylic acid (8.34%) and spermidine + salicylic acid (7. 91%); 15 days: spermidine (8.01%), salicylic acid (7.71%), and spermidine + salicylic acid (7.58%)] showed significantly higher amounts of SSC than the control group fruits (SSC amounts at different storage times; 5 days: 6.97%, 10 days: 7.30%, 15 days: 6.62%). In addition, in the study, among the different treatments according to the storage periods, the highest amount of SSC was observed in salicylic acid‐treated fruits at the 5 days storage period, while spermidine‐treated fruits were found at the 10 and 15 days storage periods (Table 1, Figure 1B).

Significant differences (*p* < 0.001) were found in TA values between treatments and the control during raspberry storage (Table 1, Figure 1C). After 5 days, fruits treated with spermidine (0.91%) and spermidine + salicylic acid (0.90%) had lower TA than the control (0.96%), while salicylic acid‐treated fruits had similar values (0.94%). By day 10, spermidine (0.80%) and salicylic acid (0.85%) treatments resulted in higher TA than the control (0.71%), while the combined treatment was similar (0.74%). After 15 days, salicylic acid‐treated fruits showed higher TA (0.78%); the combined treatment showed lower (0.68%), and spermidine treatment remained similar (0.74%) to the control (0.71%).

Significant differences (*p* < 0.001) were observed in the pH values of raspberry fruits between the control and treated groups during storage. All treatment applications—spermidine, salicylic acid, and their combination—resulted in lower pH values compared to the control at each storage interval (5, 10, and 15 days). Among them, the combined treatment of spermidine and salicylic acid consistently exhibited the lowest pH values throughout the storage period (Table 1, Figure 1D).

### 3.2 Decay and Respiration Rate

**FIGURE 2:**
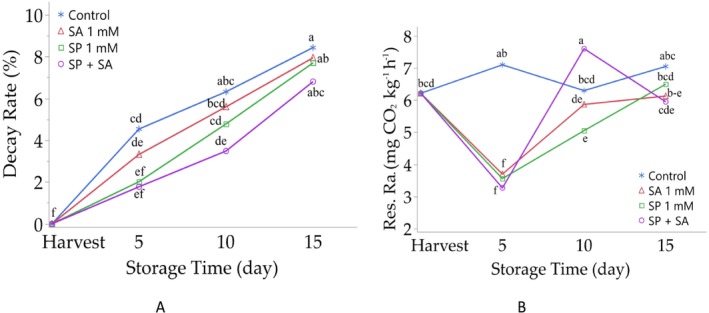
Effect of postharvest spermidine and salicylic acid application on decay rate (A) and Res. Ra.: respiration rate (B) of raspberry fruits during storage. Different letters on top of the storage periods indicate significant differences at *p* ≤ 0.05.

The differences between the decay rates of the control and treatments during storage of raspberry fruits were significant (*p* < 0.001). In the study, decay rates following all the applied treatments throughout the storage period [decay rates in different storage periods; 5 days: spermidine (2.02%), salicylic acid (3.32%) and spermidine + salicylic acid (1.77%); 10 days: spermidine (4.78%), salicylic acid (5.60%) and spermidine + salicylic acid (3. 48%); 15 days: spermidine (7.70%), salicylic acid (7.94%) and spermidine + salicylic acid (6.80%)] showed significantly lower decay rates than the control group fruits (decay rates at different storage periods; 5 days: 4.54%, 10 days: 6.32%, 15 days: 8.43%). In addition, the lowest decay rate among the different treatments used in the study at all three storage periods was found in fruits treated with spermidine + salicylic acid (Table 1, Figure 2A).

Significant differences (*p* < 0.001) in respiration rates were observed between control and treated raspberry fruits. After 5 and 15 days, all treatments (spermidine, salicylic acid, and spermidine + salicylic acid) resulted in lower respiration rates compared to the control. After 10 days, while spermidine (5.05 mg CO_2_ kg^−1^ h^−1^) and salicylic acid (5.87 mg CO_2_ kg^−1^ h^−1^) resulted in lower rates than the control (6.30 mg CO_2_ kg‐1 h‐1), the combination of spermidine + salicylic acid (7.60 mg CO_2_ kg‐1 h‐1) showed higher respiration rates than the control. Across all time points, the lowest respiration rates were observed in spermidine + salicylic acid‐treated fruits at 5 and 15 days, and in spermidine‐treated fruits at 10 days (Table 1, Figure 2B).

### 3.3 Phenolic Compounds

During the storage of raspberry fruits (5, 10 and 15 days), phenolic compounds and their amounts detected in the fruits of control and treatments were found to be significant (*p* < 0.01, *p* < 0.001). In the study, at the end of the 5‐day storage period, significantly higher amounts of phenolic compounds were observed in all different treatments [chlorogenic acid: spermidine (7.01 mg 100 g^−1^), salicylic acid (6. 75 mg 100 g^−1^) and spermidine + salicylic acid (7.25 mg 100 g^−1^); o‐coumaric acid: spermidine (1.68 mg 100 g^−1^), salicylic acid (1.58 mg 100 g^−1^) and spermidine + salicylic acid (1.71 mg 100 g^−1^); p‐coumaric acid: spermidine (1. 40 mg 100 g^−1^), salicylic acid (1.17 mg 100 g^−1^) and spermidine + salicylic acid (1.47 mg 100 g^−1^); protocatechuic acid: spermidine (1.45 mg 100 g^−1^), salicylic acid (1.41 mg 100 g^−1^) and spermidine + salicylic acid (1.47 mg 100 g^−1^)] in terms of chlorogenic acid, o‐coumaric acid, p‐coumaric acid, and protocatechuic acid compared to the control (chlorogenic acid: 6. 50 mg 100 g^−1^, o‐coumaric acid: 1.42 mg 100 g^−1^, p‐coumaric acid: 1.09 mg 100 g^−1^, protocatechuic acid: 1.31 mg 100 g^−1^) group fruits. Moreover, at the end of the same storage period, significantly higher amounts of gallic acid were detected in both spermidine and salicylic acid‐treated fruits [spermidine (1.54 mg 100 g^−1^), salicylic acid (1.35 mg 100 g^−1^)] than in the control (1.24 mg 100 g^−1^) group fruits (Tables 2 and 3).

**TABLE 2:** Impact of different doses of spermidine and salicylic acid applications on phenolic compound contents of raspberry fruits during storage (mg/100 g).

Storage time (day)	Catechin	Chlorogenic	Ferulic	Gallic	o‐Coumaric
Harvest	37.97 ± 1.22ab	7.36 ± 0.07a	1.63 ± 0.05abc	1.46 ± 0.20a	1.73 ± 0.07a
5	43.14 ± 1.88a	6.88 ± 0.11a	1.98 ± 0.08a	1.32 ± 0.06a	1.60 ± 0.05ab
10	39.13 ± 1.20ab	5.72 ± 0.16b	1.67 ± 0.09b	1.16 ± 0.05b	1.41 ± 0.05b
15	35.86 ± 1.11b	4.58 ± 0.11c	1.33 ± 0.08c	0.96 ± 0.02c	1.18 ± 0.09c
SP and SA × Storage time interaction					
Harvest	37.97 ± 1.22bcde	7.36 ± 0.07a	1.63 ± 0.05 cdef	1.46 ± 0.20ab	1.73 ± 0.07a
Day 5					
Control	50.57 ± 1.84a	6.50 ± 0.08de	2.26 ± 0.08a	1.25 ± 0.02bcd	1.42 ± 0.06bc
SP1mM	41.30 ± 2.45b	7.01 ± 0.19bc	1.92 ± 0.08bc	1.54 ± 0.02a	1.68 ± 0.08a
SA1mM	41.71 ± 3.39b	6.75 ± 0.04 cd	1.69 ± 0.03cde	1.35 ± 0.03abc	1.58 ± 0.11ab
SP + SA (1 mM)	39.01 ± 0.91bcd	7.25 ± 0.05ab	2.04 ± 0.10ab	1.13 ± 0.10cde	1.71 ± 0.07a
Day 10					
Control	35.77 ± 2.78cde	5.36 ± 0.03 g	1.77 ± 0.11bcd	1.15 ± 0.16cde	1.26 ± 0.05c
SP1mM	42.27 ± 2.20b	5.79 ± 0.04f	1.55 ± 0.23defg	1.16 ± 0.07cde	1.44 ± 0.06bc
SA1mM	40.59 ± 0.63bc	5.38 ± 0.17 g	1.44 ± 0.08efg	1.29 ± 0.01abc	1.38 ± 0.07bc
SP + SA (1 mM)	37.91 ± 1.62bcde	6.37 ± 0.07e	1.91 ± 0.11bc	1.02 ± 0.08de	1.56 ± 0.05ab
Day 15					
Control	33.26 ± 1.14e	4.23 ± 0.04ı	1.28 ± 0.04gh	0.97 ± 0.08e	0.79 ± 0.04d
SP1mM	35.81 ± 0.30cde	4.71 ± 0.04 h	1.34 ± 0.08fgh	0.97 ± 0.01e	1.24 ± 0.08c
SA1mM	40.53 ± 0.50bc	4.44 ± 0.08ı	1.08 ± 0.06 h	1.01 ± 0.01de	1.29 ± 0.06c
SP + SA (1 mM)	33.85 ± 0.77de	4.96 ± 0.03 h	1.64 ± 0.06cdef	0.91 ± 0.05e	1.39 ± 0.07bc
ANOVA					
*F* storage time	4.51*	10.76***	68.32***	1.71^ns^	22.14***
*F* storage time × SP and SA	6.44**	157.83***	11.11***	5.24**	13.36***

*Note:* Different letters in the same column indicates statistical differences at *p* ≤ 0.05. ns: not significant. *, **, *** indicates *p* ≤ 0.05, 0.01, and 0.001, respectively.

**TABLE 3:** Continue Table 2 (mg/100 g).

Storage time (day)	p‐Coumaric	Protocatechuic	Quercetin	Rutin
Harvest	1.48 ± 0.06a	1.70 ± 0.02a	1.34 ± 0.04abc	7.22 ± 0.35b
5	1.28 ± 0.07ab	1.41 ± 0.03b	1.62 ± 0.07a	11.10 ± 0.94ab
10	1.14 ± 0.07bc	1.29 ± 0.04c	1.37 ± 0.07b	12.46 ± 0.76a
15	1.01 ± 0.06c	1.15 ± 0.04d	1.09 ± 0.06c	12.34 ± 1.55ab
SP and SA × Storage time interaction				
Harvest	1.48 ± 0.06a	1.70 ± 0.02a	1.34 ± 0.04 cdef	7.22 ± 0.35d
Day 5				
Control	1.09 ± 0.10de	1.31 ± 0.03de	1.86 ± 0.06a	14.10 ± 0.46abc
SP1mM	1.40 ± 0.08ab	1.45 ± 0.05b	1.57 ± 0.07bc	9.50 ± 1.00bcd
SA1mM	1.17 ± 0.07bcd	1.41 ± 0.03bc	1.39 ± 0.02cde	8.10 ± 0.40 cd
SP + SA (1 mM)	1.47 ± 0.09a	1.47 ± 0.02b	1.67 ± 0.08ab	12.70 ± 0.70a‐d
Day 10				
Control	0.98 ± 0.09de	1.17 ± 0.02 g	1.45 ± 0.09bcd	14.85 ± 0.64ab
SP1mM	1.13 ± 0.14 cd	1.34 ± 0.01 cd	1.28 ± 0.18d‐g	11.24 ± 0.99a‐d
SA1mM	1.08 ± 0.10de	1.24 ± 0.02efg	1.18 ± 0.07efg	10.20 ± 0.88abcd
SP + SA (1 mM)	1.37 ± 0.09abc	1.41 ± 0.03bc	1.56 ± 0.09bc	13.57 ± 0.64abc
Day 15				
Control	0.84 ± 0.05e	1.02 ± 0.02ı	1.05 ± 0.03 gh	11.19 ± 0.93a‐d
SP1mM	1.09 ± 0.06de	1.23 ± 0.02 fg	1.10 ± 0.06fgh	15.90 ± 6.58a
SA1mM	0.93 ± 0.08de	1.09 ± 0.03 h	0.89 ± 0.05 h	12.68 ± 1.15a‐d
SP + SA (1 mM)	1.19 ± 0.07bcd	1.26 ± 0.03ef	1.34 ± 0.05 cde	9.61 ± 0.76bcd
ANOVA				
*F* storage time	5.2**	8.89***	10.41***	10.36***
*F* storage time × SP and SA	5.65**	53.29***	11.26***	1.79**

*Note:* Different letters in the same column indicate statistical differences at *p* ≤ 0.05. ns: not significant. *, **, *** indicate *p* ≤ 0.05, 0.01, and 0.001, respectively.

In the study, at the end of the 10‐day storage period, significantly higher amounts of phenolic compounds were observed in all different treatments [catechin: spermidine (42.27 mg 100 g^−1^), salicylic acid (40.59 mg 100 g^−1^) and spermidine + salicylic acid (37. 91 mg 100 g^−1^); o‐coumaric acid: spermidine (1.44 mg 100 g^−1^), salicylic acid (1.38 mg 100 g^−1^) and spermidine + salicylic acid (1.56 mg 100 g^−1^); protocatechuic acid: spermidine (1.34 mg 100 g^−1^), salicylic acid (1.24 mg 100 g^−1^) and spermidine + salicylic acid (1.41 mg 100 g^−1^)] in terms of catechin, o‐coumaric acid, and protocatechuic acid compared to the control (catechin: 35.77 mg 100 g^−1^, o‐coumaric acid: 1.26 mg 100 g^−1^, protocatechuic acid: 1.17 mg 100 g^−1^) group fruits. Moreover, at the end of the same storage period, significantly higher amounts of chlorogenic and p‐coumaric acids were detected in spermidine and spermidine + salicylic acid‐treated fruits [chlorogenic acid: spermidine (5.79 mg 100 g^−1^), spermidine + salicylic acid (6.37 mg 100 g^−1^); p‐coumaric acid: spermidine (1.13 mg 100 g^−1^), spermidine + salicylic acid (1.37 mg 100 g^−1^)], ferulic acid and quercetin in spermidine + salicylic acid‐treated fruits (ferulic acid: 1.91 mg 100 g^−1^, quercetin: 1.56 mg 100 g^−1^), and gallic acid in salicylic acid‐treated fruits (1.29 mg 100 g^−1^) compared to the control (catechin: 35.77 mg 100 g^−1^, chlorogenic acid: 5.36 mg 100 g^−1^, o‐coumaric acid: 1.26 mg 100 g^−1^, ferulic acid: 1.77 mg 100 g^−1^, gallic acid: 1.15 mg 100 g^−1^, p‐coumaric acid: 0.98 mg 100 g^−1^, protocatechuic acid: 1.17 mg 100 g^−1^, quercetin: 1.45 mg 100 g^−1^) group fruits (Tables 2 and 3).

In the study, at the end of the 15‐day storage period, significantly higher amounts of phenolic compounds were observed in all different treatments [catechin: spermidine (35.81 mg 100 g^−1^), salicylic acid (40.53 mg 100 g^−1^) and spermidine + salicylic acid (33.85 mg 100 g^−1^); o‐coumaric acid: spermidine (1.24 mg 100 g^−1^), salicylic acid (1.29 mg 100 g^−1^) and spermidine + salicylic acid (1.39 mg 100 g^−1^); p‐coumaric acid: spermidine (1.09 mg 100 g^−1^), salicylic acid (0.93 mg 100 g^−1^) and spermidine + salicylic acid (1.19 mg 100 g^−1^); protocatechuic acid: spermidine (1.23 mg 100 g^−1^), salicylic acid (1.09 mg 100 g^−1^) and spermidine + salicylic acid (1.26 mg 100 g^−1^)] in terms of catechin, o‐coumaric acid, p‐coumaric acid and protocatechuic acid compared to the control (catechin: 33.26 mg 100 g^−1^, o‐coumaric acid: 0.79 mg 100 g^−1^, p‐coumaric acid: 0.84 mg 100 g^−1^, protocatechuic acid: 1.02 mg 100 g^−1^) group fruits. Moreover, at the end of the same storage period, significantly higher amounts of chlorogenic acid, ferulic acid and quercetin were detected in spermidine and spermidine + salicylic acid‐treated fruits [chlorogenic acid: spermidine (4.71 mg 100 g^−1^), spermidine + salicylic acid (4.96 mg 100 g^−1^); ferulic acid: spermidine (1.34 mg 100 g^−1^), spermidine + salicylic acid (1.64 mg 100 g^−1^); quercetin: spermidine (1.10 mg 100 g^−1^), spermidine + salicylic acid (1.34 mg 100 g^−1^)], gallic acid in salicylic acid‐treated fruits (1.01 mg 100 g^−1^) and rutin in spermidine‐treated fruits (15.90 mg 100 g^−1^) compared to the control (chlorogenic acid: 4.23 mg 100 g^−1^, ferulic acid: 1.28 mg 100 g^−1^, quercetin: 1.05 mg 100 g^−1^, gallic acid: 0.97 mg 100 g^−1^, routine: 11.19 mg 100 g^−1^) group fruits (Tables 2 and 3).

Accordingly, among the different treatments used in the study, spermidine + salicylic acid treatment generally prevented phenolic compound losses more than the control fruits (Tables 2 and 3). Furthermore, the fact that the different treated fruits contained significantly higher amounts of certain phenolic compounds than the control fruits to a varying extent over different storage periods indicates that phenolic compounds declined more slowly in the treated fruits. Hence, this was attributed to the fact that the different treatments used prevented the loss of phenolic compounds in the fruits. In addition, it was determined that catechin was the most abundant phenolic compound in raspberry fruits, followed by rutin and chlorogenic acid compounds, respectively. Accordingly, catechin, rutin, and chlorogenic acid contents were determined as 35.86 mg 100 g^−1^, 12.34 mg 100 g,^−1^ and 4.58 mg 100 g^−1^, respectively, at the end of 15 days storage (Tables 2 and 3).

### 3.4 Organic Acids and Vitamin C

During the storage of raspberry fruits (5, 10 and 15 days), organic acids and vitamin C and their amounts in the fruits of control and treatments were found to be significant (*p* < 0.001).

In the study, at the end of the 5‐day storage period, significantly higher organic acid amounts were observed in all different treatments [citric acid: spermidine (977.80 mg 100 g^−1^), salicylic acid (933.58 mg 100 g^−1^) and spermidine + salicylic acid (1004.56 mg 100 g^−1^); malic acid: spermidine (207.05 mg 100 g^−1^), salicylic acid (188.55 mg 100 g^−1^) and spermidine + salicylic acid (189.88 mg 100 g^−1^)] in terms of citric acid and malic acid compared to the control (citric acid: 879.12 mg 100 g^−1^, malic acid: 182.33 mg 100 g^−1^) group fruits. In addition, at the end of the same storage period, significantly higher amounts of oxalic acid were detected in spermidine and spermidine + salicylic acid‐treated fruits [spermidine (13.14 mg 100 g^−1^), spermidine + salicylic acid (14.62 mg 100 g^−1^)], succinic acid in spermidine‐treated fruits (12.24 mg 100 g^−1^), and fumaric acid in spermidine + salicylic acid‐treated fruits (4.86 mg 100 g^−1^) compared to the control (succinic acid: 11.28 mg 100 g^−1^, oxalic acid: 12.50 mg 100 g^−1^, fumaric acid: 4.66 mg 100 g^−1^) group fruits. In addition, at the end of this storage period, all of the different treatments [spermidine (24.92 mg 100 g^−1^), salicylic acid (20.72 mg 100 g^−1^) and spermidine + salicylic acid (26.04 mg 100 g^−1^)] had significantly higher amounts of vitamin C than the control (18.93 mg 100 g^−1^) group fruits (Table 4).

**TABLE 4:** Impact of different doses of spermidine and salicylic acid applications on organic acid and vitamin C contents of raspberry fruits during storage (mg/100 g).

Storage time (day)	Citric	Malic	Succinic	Oxalic	Fumaric	Vitamin C
Harvest	1017.09 ± 7.51a	213.61 ± 1.39a	14.58 ± 0.08a	15.36 ± 0.53a	5.10 ± 0.02a	25.87 ± 0.55a
5	948.76 ± 18.31a	191.95 ± 3.72b	11.42 ± 0.23b	13.20 ± 0.36b	4.62 ± 0.10a	22.65 ± 1.15a
10	845.63 ± 21.89b	162.74 ± 5.63c	10.16 ± 0.31c	12.01 ± 0.32c	4.09 ± 0.14b	21.29 ± 0.94a
15	703.45 ± 15.40c	134.81 ± 4.51d	9.35 ± 0.46c	10.89 ± 0.34d	3.65 ± 0.10c	16.13 ± 0.96b
SP and SA x Storage time interaction						
Harvest	1017.09 ± 7.51a	213.61 ± 1.39a	14.58 ± 0.08a	15.36 ± 0.53a	5.10 ± 0.02a	25.87 ± 0.55a
Day 5						
Control	879.12 ± 7.13d	182.33 ± 2.39bc	11.28 ± 0.56c	12.50 ± 0.28bcd	4.66 ± 0.32abc	18.93 ± 0.68 cd
SP1mM	977.80 ± 13.18b	207.05 ± 5.31a	12.24 ± 0.27b	13.14 ± 0.53b	4.51 ± 0.18bc	24.92 ± 0.59a
SA1mM	933.58 ± 5.93c	188.55 ± 3.02b	11.22 ± 0.22 cd	12.54 ± 0.35bcd	4.44 ± 0.18bc	20.72 ± 1.31bc
SP + SA (1 mM)	1004.56 ± 9.68ab	189.88 ± 2.67b	10.94 ± 0.18 cd	14.62 ± 0.44a	4.86 ± 0.08ab	26.04 ± 0.67a
Day 10						
Control	755.49 ± 9.14f	140.96 ± 4.14e	8.80 ± 0.10f	10.72 ± 0.26ef	3.89 ± 0.16de	17.67 ± 0.89de
SP1mM	870.49 ± 7.84de	175.42 ± 0.89c	10.92 ± 0.18 cd	12.53 ± 0.31bcd	4.52 ± 0.17bc	21.50 ± 0.72b
SA1mM	844.90 ± 12.30e	157.65 ± 1.85d	10.45 ± 0.17d	11.93 ± 0.18 cd	3.70 ± 0.28ef	21.68 ± 0.53b
SP + SA (1 mM)	911.66 ± 4.35c	176.94 ± 1.68c	10.48 ± 0.19d	12.86 ± 0.22bc	4.26 ± 0.15 cd	24.30 ± 0.88a
Day 15						
Control	638.29 ± 11.49 h	121.68 ± 3.92f	7.63 ± 0.20 g	9.92 ± 0.28f	3.23 ± 0.11f	12.07 ± 0.59f
SP1mM	726.71 ± 12.66 fg	139.82 ± 1.87e	11.00 ± 0.33 cd	11.61 ± 0.41de	3.75 ± 0.14def	17.77 ± 0.75de
SA1mM	709.87 ± 10.11 g	126.39 ± 1.87f	9.60 ± 0.18e	10.23 ± 0.30f	3.72 ± 0.11ef	16.38 ± 0.86e
SP + SA (1 mM)	738.94 ± 12.69 fg	151.37 ± 2.14d	9.17 ± 0.31ef	11.79 ± 0.43cde	3.89 ± 0.08de	18.30 ± 0.39de
ANOVA						
*F* storage time	37.97***	35.29***	18.26***	15.31***	17.93***	10.53***
*F* Storage time × SP and SA	153.16***	111.48***	43.34***	18.45***	10.14***	30.07***

*Note:* Different letters in the same column indicate statistical differences at *p* ≤ 0.05. *, **, *** indicate *p* ≤ 0.05, 0.01, and 0.001, respectively.

In the study, at the end of the 10‐day storage period, significantly higher organic acid amounts were observed in all different treatments [citric acid: spermidine (870.49 mg 100 g^−1^), salicylic acid (844.90 mg 100 g^−1^) and spermidine + salicylic acid (911.66 mg 100 g^−1^); malic acid: spermidine (175.42 mg 100 g^−1^), salicylic acid (157.65 mg 100 g^−1^) and spermidine + salicylic acid (176.94 mg 100 g^−1^); succinic acid: spermidine (10.92 mg 100 g^−1^), salicylic acid (10.45 mg 100 g^−1^) and spermidine + salicylic acid (10.48 mg 100 g^−1^); oxalic acid: spermidine (12.53 mg 100 g^−1^), salicylic acid (11.93 mg 100 g^−1^) and spermidine + salicylic acid (12.86 mg 100 g^−1^)] in terms of citric, malic, succinic, and oxalic acids compared to the control (citric acid: 755.49 mg 100 g^−1^, malic acid: 140.96 mg 100 g^−1^, succinic acid: 8.80 mg 100 g^−1^, oxalic acid: 10.72 mg 100 g^−1^) group fruits. In addition, at the end of the same storage period, significantly higher amounts of fumaric acid were detected in spermidine and spermidine + salicylic acid‐treated fruits [spermidine (4.52 mg 100 g^−1^), spermidine + salicylic acid (4.26 mg 100 g^−1^)] than in control (3.89 mg 100 g^−1^) group fruits. Moreover, at the end of this storage period, all of the different treatments [spermidine (21.50 mg 100 g^−1^), salicylic acid (21.68 mg 100 g^−1^) and spermidine + salicylic acid (24.30 mg 100 g^−1^)] had significantly higher amounts of vitamin C compared to the control (17.67 mg 100 g^−1^) group fruits (Table 4).

In the study, at the end of the 15‐day storage period, significantly higher organic acid amounts were observed in all different treatments [citric acid: spermidine (726.71 mg 100 g^−1^), salicylic acid (709.87 mg 100 g^−1^) and spermidine + salicylic acid (738.94 mg 100 g^−1^); succinic acid: spermidine (11.00 mg 100 g^−1^), salicylic acid (9.60 mg 100 g^−1^) and spermidine + salicylic acid (9.17 mg 100 g^−1^); fumaric acid: spermidine (3.75 mg 100 g^−1^), salicylic acid (3.72 mg 100 g^−1^) and spermidine + salicylic acid (3.89 mg 100 g^−1^)] in terms of citric, succinic, and fumaric acids compared to the control (citric acid: 638.29 mg 100 g^−1^, succinic acid: 7.63 mg 100 g^−1^, fumaric acid: 3.23 mg 100 g^−1^) group fruits. In addition, at the end of the same storage period, significantly higher amounts of malic and oxalic acids were detected in spermidine and spermidine + salicylic acid‐treated fruits [malic acid: spermidine (139.82 mg 100 g^−1^), spermidine + salicylic acid (151.37 mg 100 g^−1^); oxalic acid: spermidine (11.61 mg 100 g^−1^), spermidine + salicylic acid (11.79 mg 100 g^−1^)] than in the control (malic acid: 121.68 mg 100 g^−1^, oxalic acid: 9.92 mg 100 g^−1^) group fruits. Moreover, at the end of this storage period, all of the different treatments [spermidine (17.77 mg 100 g^−1^), salicylic acid (16.38 mg 100 g^−1^) and spermidine + salicylic acid (18.30 mg 100 g^−1^)] had significantly higher amounts of vitamin C than the control (12.07 mg 100 g^−1^) group fruits (Table 4).

Accordingly, it was determined that, in general, among the different treatments used, both spermidine and spermidine + salicylic acid treatments prevented organic acid losses more than the control fruits, although it varied according to different storage periods. In addition, vitamin C was significantly better preserved in all treated fruits than in control fruits. Furthermore, citric acid was found to be the most dominant organic acid in raspberry fruits, followed by malic acid and vitamin C, respectively. Accordingly, citric acid, malic acid, and vitamin C contents were determined as 703.45 mg 100 g^−1^, 134.81 mg 100 g,^−1^ and 16.13 mg 100 g^−1^, respectively, at the end of 15 days storage (Table 4).

### 3.5 Principal Component Analysis and HeatMap Analysis

**FIGURE 3:**
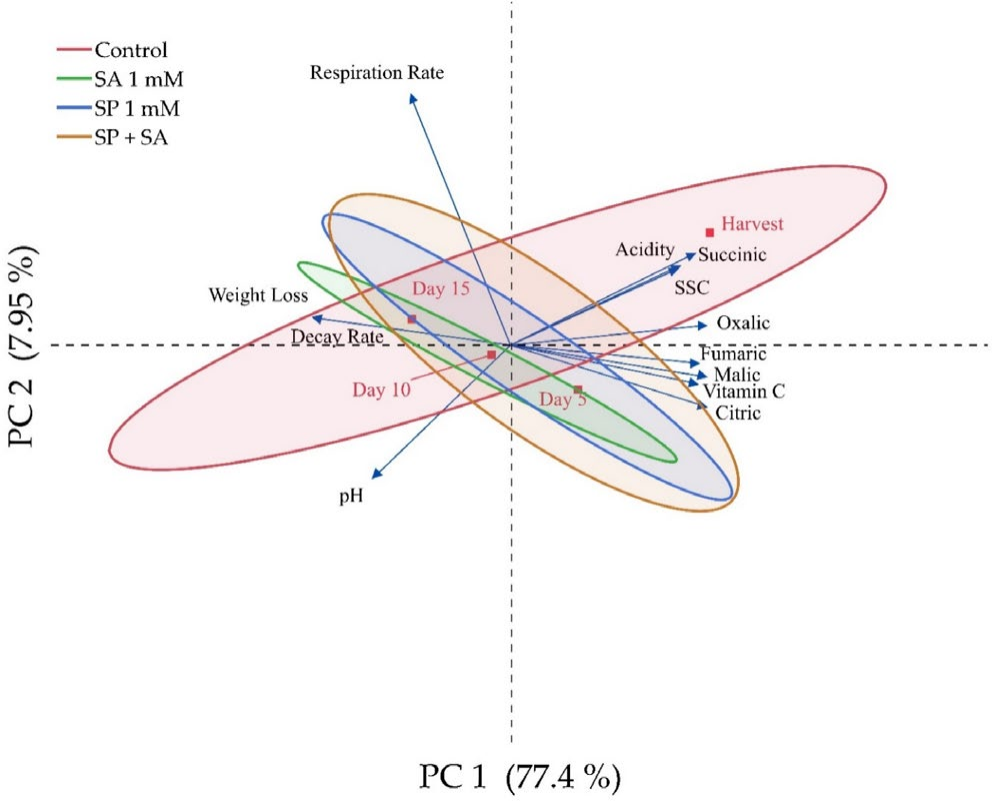
Determination of the effects of spermidine and salicylic acid applications on changes in organic acid and quality properties of fruits during storage by PCA analysis.

This study was conducted to determine the effects of spermidine and salicylic acid treatments on the changes of organic acid and quality characteristics of raspberry fruits during postharvest storage by PCA analysis (Figure 3). PCA analysis is an effective method to determine which characteristics are more critical and explanatory in the findings. In addition, PCA analysis is also important in terms of producing effective results by minimizing the number of factors when separating data in studies (Gundogdu 2019; Gecer et al. 2020; Kaskoniene et al. 2020). Both agromorphological characteristics and organic acid content of raspberry fruits during cold storage showed variation in the effects of spermidine and salicylic acid treatments depending on the dose applied. It was also observed that the total variation was significantly explained by the first two principal component axes with a value of 85.35%. The first principal component axis accounted for 77.4% of the total variation and the second principal component axis accounted for 7.95% of the total variation. These axes were found to be important in the evaluation of the analysis. In the two‐dimensional graph, it was determined that there were differences between the treatments in terms of the parameters analyzed. The control group and spermidine + salicylic acid treatments provided a wider range of physicochemical and organic acids, while the other treatments provided a narrower range. Spermidine and spermidine + salicylic acid treatments provided a wider range, especially for organic acids. Succinic, oxalic, fumaric, malic, vitamin C, and citric acid values were negatively correlated with weight loss, respiration rate, pH, and decay rate values. In addition, fumaric, malic, vitamin C, and citric acid values were parallel and in the same plane.

**FIGURE 4:**
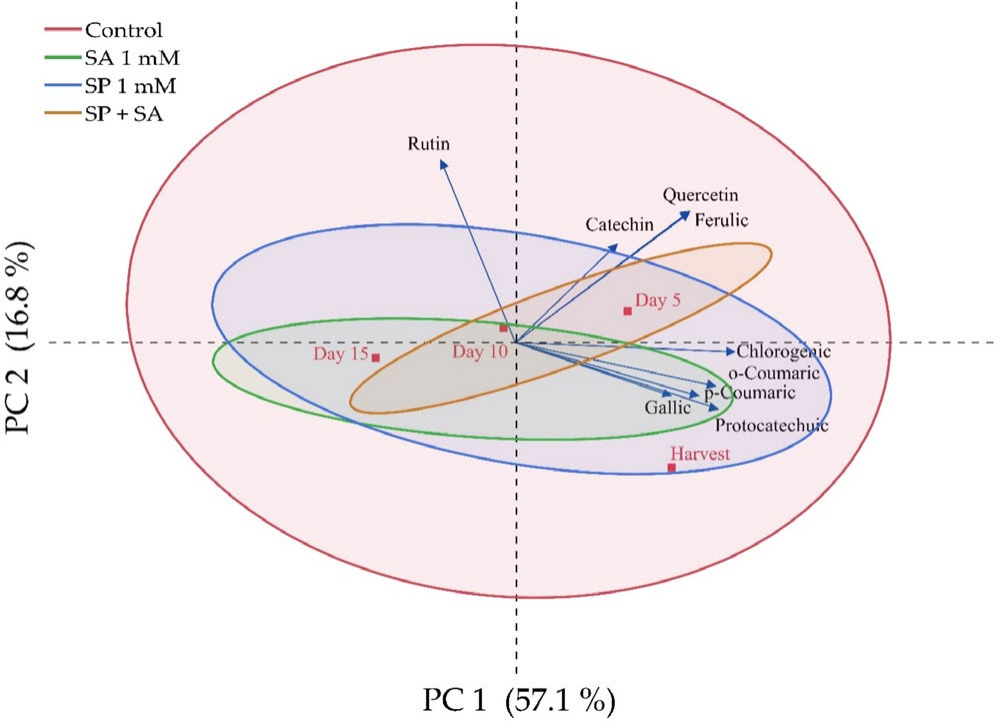
Determination of the effects of spermidine and salicylic acid applications on changes in phenolic compounds of fruits during storage by PCA analysis.

In this study, PCA was performed to determine the effect of spermidine and salicylic acid doses on the phenolic compound contents of raspberry fruits and the correlation of phenolic compounds with each other during postharvest storage (Figure 4). The results showed that the total variation was significantly explained by the first two principal component axes with a value of 73.9%. The first principal component axis accounts for 57.1% of the total variation, and the second principal component axis accounts for 16.8% of the total variation. These axes were found to be significant in the evaluation of the analysis. In the two‐dimensional graph, it was determined that there were differences between the varieties in terms of phenolic compounds. Spermidine treatment and the control group provided a wide range of phenolic compounds, while other treatments provided a narrower range. It was observed that chlorogenic, o‐coumaric, p‐coumaric, gallic, and protocatechuic values were in parallel with each other and were more abundant in salicylic acid, spermidine, and the control group. Similarly, catechin, quercetin, and ferulic values were in parallel with each other and were found to be higher in spermidine + salicylic acid, spermidine, and the control group treatments compared to other treatments. Rutin value was found to have a negative relationship with other phenolic compound values.

**FIGURE 5:**
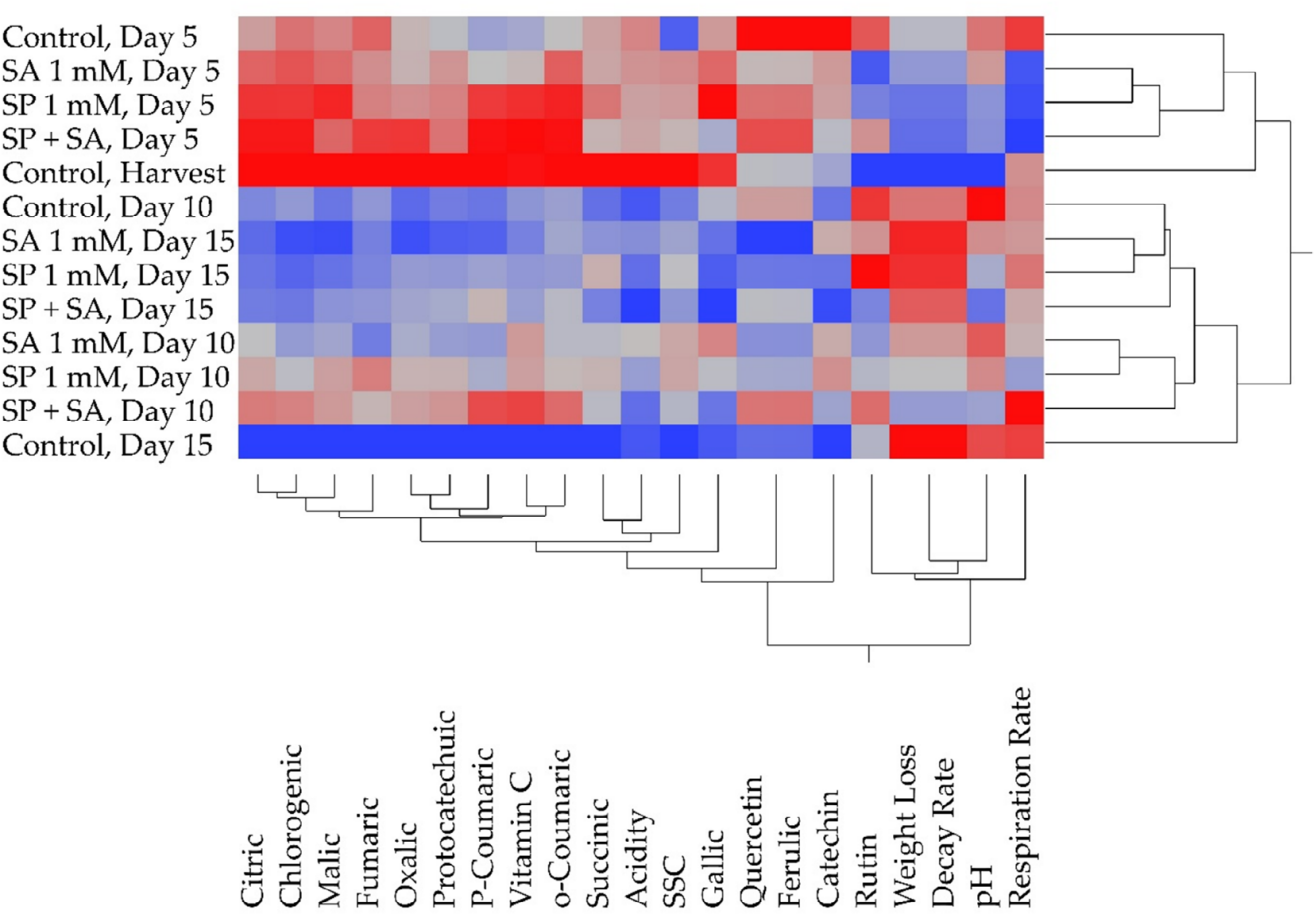
Identification of the relationship between changes in quality and biochemical properties of fruits during storage using heatmap analysis.

In the hierarchical clustering analysis, raspberry fruits were divided into different clusters. In heat mapping analysis, the color change towards red in the color scale indicates an increase in the level of statistical significance. In the study, heat map analysis was used to determine the relationship between changes in quality and biochemical properties of raspberry fruits during storage. According to the heat map results, day 5 spermidine treatment was separated from the other groups with higher citric, chlorogenic, malic, p‐coumaric, vitamin C, and o‐coumaric acids. Similarly, the significance levels of citric, chlorogenic, fumaric, oxalic, p‐coumaric, vitamin C, and o‐coumaric acid values were higher with spermidine + salicylic acid application on the 5th day. Similarly, higher citric, chlorogenic, malic, fumaric, oxalic, protocatechuic, p‐coumaric, vitamin C, and o‐coumaric acid values were found in control and harvest groups. Vitamin C and o‐coumaric, succinic, acidity, SSC, and gallic acid values formed a separate cluster from the others. Day 15 and the control group were found to be lower than other biochemical compounds in terms of citric, chlorogenic, malic, fumaric, oxalic, protocatechuic, p‐coumaric, vitamin C, and o‐coumaric, succinic, acidity, SSC, and gallic, quercetin, ferulic, and catechin values and formed a separate cluster. However, the 15th day and control group weight loss, decay rate, pH, and respiration rate values were found to be statistically significant (Figure 5).

**FIGURE 6:**
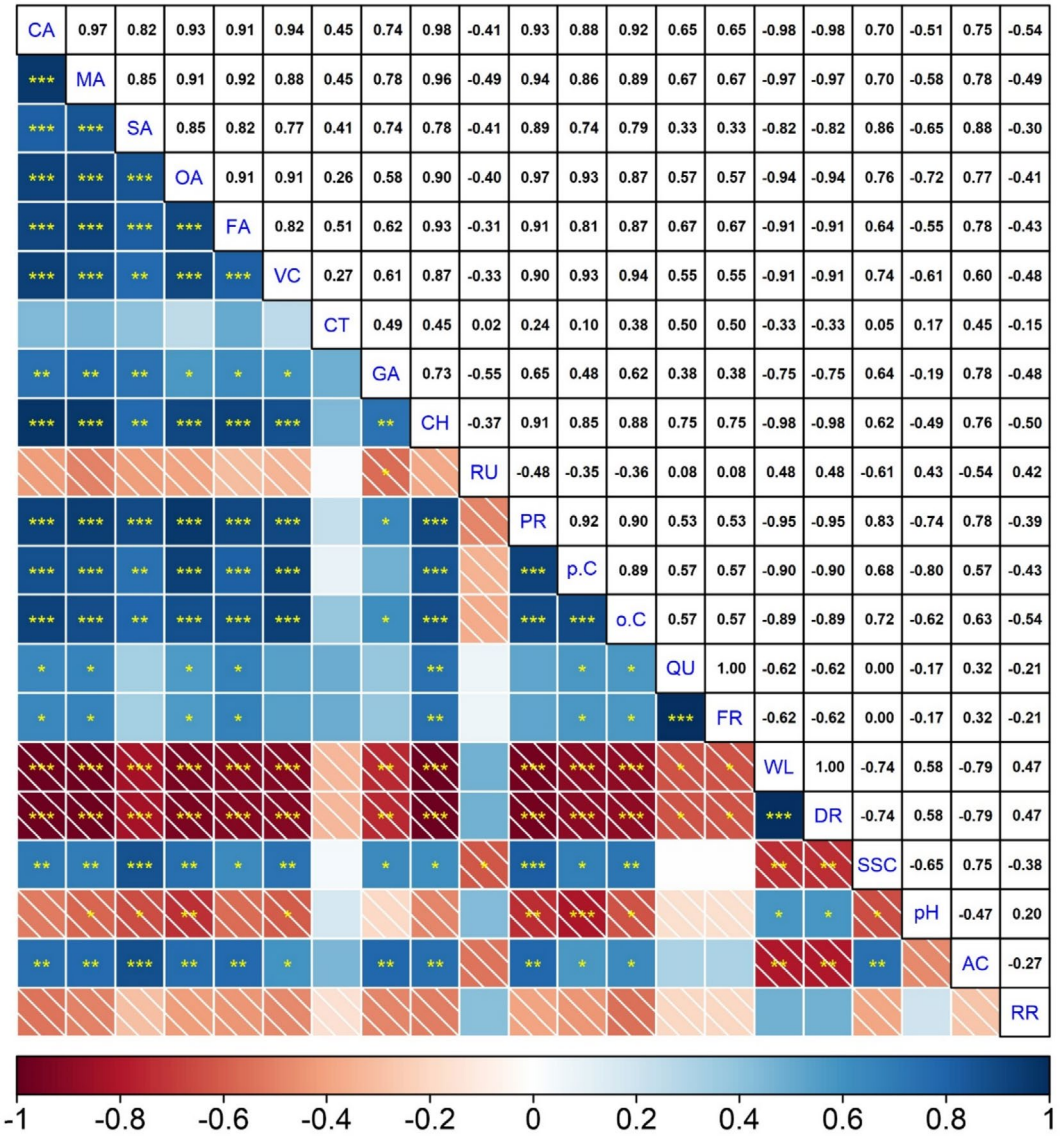
Intercourse among quality and chemical properties of raspberry fruits. The color gradient ranging from red to blue represents correlation values between −1 and +1. *, **, and *** denote significance levels at *p* ≤ 0.05, *p* ≤ 0.01, and *p* ≤ 0.001, respectively. AC, acidity; CA, citric acid; CH, chlorogenic; CT, catechin; DR, decay rate; FA, fumaric acid; GA, gallic; MA, malic acid; *o.C*, *O*‐coumaric; OA, oxalic; *p.C*, *P*‐coumaric; PR, protocatechuic; QU, quercetin; FR, ferulic; RR, respiration rate; RU, rutin; SA, succinic acid; SSC, soluble solids content; VC, vitamin C; WL, weight loss.

Citric acid was positively correlated with malic acid (*r* = 0.97, *p* ≤ 0.001), while vitamin C was negatively correlated with respiration rate (*r* = −0.48, *p* ≤ 0.001), pH (*r* = −0.61), weight loss (*r* = − 0.91) and decay rate (*r* = −0.91). Organic acids were highly positively correlated with each other, while phenolic compounds showed a similar high positive correlation among themselves (Figure 6). Citric acid showed a positive correlation with malic acid (*r* = 0.97, *p* ≤ 0.001), succinic acid (*r* = 0.82, *p* ≤ 0.001), oxalic acid (*r* = 0.93, *p* ≤ 0.001), fumaric acid (*r* = 0.91, *p* ≤ 0.001), gallic acid (*r* = 0.74, *p* ≤ 0.001) and chlorogenic acid (*r* = 0.98, *p* ≤ 0.001). Similarly, vitamin C showed a positive correlation with phenolic compounds and organic acids, especially with ascorbic acid, citric acid (*r* = 0.94, *p* ≤ 0.001), oxalic acid (*r* = 0.91, *p* ≤ 0.001) and malic acid (*r* = 0.88, *p* ≤ 0.001).

## 4 Discussion

In this study, there are examples in the literature for various fruits that support results on weight loss in raspberries. Orman (2023) observed significantly less weight loss in nectarines of the ‘Fantasia’ variety treated with 1.5 mM spermidine and stored for 30 days. Jalali et al. (2023) reported that weight loss was the lowest in strawberries stored for 12 days and treated with 1.0 and 1.5 mM spermidine. Gündoğdu et al. (2023) reported that plum stored for 20 and 40 days and treated with 1.5 mM spermidine showed less weight loss than control fruits. Physiological weight loss usually occurs due to various metabolic activities such as respiration and transpiration. Indeed, in this study, all the different treatments used in all three storage periods significantly reduced weight loss compared to the control. Therefore, this may be related to the lower respiration rates detected in spermidine and salicylic acid‐treated fruits (Table 1). Furthermore, a number of researchers have reported that the application of polyamines such as spermidine in various stored fruits improves membrane fluidity in fruits, improves membrane health, prevents water loss and thus better prevents weight loss (Mirdehghan et al. 2007; Zahedi et al. 2019).

Orman (2023) observed significantly lower amounts of SSC in nectarine fruits treated with 1.0 and 1.5 mM spermidine and stored for 30 days compared to control fruits. Jalali et al. (2023) reported that in strawberries stored for 12 days and treated with different doses of spermidine, lower amounts of SSC were observed in fruits treated with 1.5 mM spermidine compared to control fruits. Hosseini et al. (2018) stated that in pears stored for different periods of time, the application of polyamines such as spermidine significantly increased the amount of SSC compared to control fruits. Accordingly, when the results of the literature studies and the results of this study were evaluated together, partly similar and partly opposite results were obtained. Regarding the increase in SSC in stored fruits with the application of polyamines such as spermidine, some researchers have stated that the applied polyamines delay softening and ethylene synthesis in fruits more and accordingly, increases in quality parameters such as SSC, vitamin C, and phenolic compound contents (Hosseini et al. 2018).

Sabir et al. (2019) treated blackberry fruits with 2 mM salicylic acid and stored them for 4, 7, and 10 days. They observed that the TA values were higher in the salicylic acid‐treated fruits compared to the control group for all storage durations. Similar to this literature, in this study, in raspberry fruits stored for 10 and 15 days, only salicylic acid‐treated fruits had significantly higher TA values than control fruits. In addition, some researchers reported that polyamine treatments such as spermidine increased titratable acidity value in various fruits stored for certain periods (Davarynejad et al. 2013; Mirdehghan and Rahimi 2016). Similar to this statement, in this study, raspberry fruits stored for 10 days showed significantly higher TA values only in spermidine‐treated fruits compared to control fruits. Accordingly, the researchers stated that the higher TA values in stored polyamine‐treated fruits were due to the fact that polyamine treatment significantly reduced the respiration rate and ethylene biosynthesis processes, thereby better preserving fruit titratable acidity (Davarynejad et al. 2013).

Similar to raspberry data, Orman (2023) observed significantly lower pH values in nectarine fruit stored for 30 days and treated with different spermidine doses compared to control fruits. Accordingly, in line with the findings of the studies, Davarynejad et al. (2013) stated that spermidine application in stored fruits decreased the respiration rate and ethylene synthesis, thus preserving the titratable acidity of the fruit more, thereby decreasing the pH values, which have an inversely proportional relationship with titratable acidity.

Similar to the decay rate results detected in this study, Orman (2023) reported that nectarines stored for 30 days and treated with a 1.0 mM spermidine dose showed significantly less decay compared to control fruits. Jalali et al. (2023) reported that in strawberries treated with different doses of spermidine, the lowest decay rate was observed in fruits treated with 1.5 mM spermidine after 6 and 12 days of storage. Therefore, according to the findings obtained in studies related to decay rate, Davarynejad et al. (2013) reported that polyamine application to stored fruits slowed down the fruit ripening rate by reducing respiration rate and ethylene synthesis, and thus, decay activity can be significantly delayed in fruits with a slower ripening rate.

Similar to findings in terms of respiration rate, Orman (2023) reported that in nectarine stored and treated with different doses of spermidine, fruits treated with 1.5 mM spermidine had a significantly lower respiration rate compared to control fruits. Gündoğdu et al. (2023) reported that in plum stored for 20 and 40 days and treated with different doses of spermidine, fruits treated with a 1.5 mM spermidine dose had a significantly lower respiration rate compared to control fruits. Accordingly, considering these findings in terms of respiration rate in various fruits in the studies, many researchers have reported a direct relationship between the slowed respiration rate and delayed fruit ripening in spermidine‐treated fruits (Razzaq et al. 2014). In addition, Hanif et al. (2020) reported that spermidine‐treated fruits excreted lower amounts of CO_2_, decreased metabolic activity, and reduced respiration rate due to these effects.

Results on phenolic compounds in raspberry were in parallel with the results of some other literature studies on this subject in various fruits. Sayyari and Valero (2012) applied 2 mM salicylic acid to stored pomegranate fruit and reported that the amount of phenolic compounds in fruits with salicylic acid was significantly higher than in control fruits. Orman (2023) reported that the most dominant phenolic compounds detected in nectarine stored for 30 days and treated with different spermidine doses were chlorogenic acid, quercetin and rutin, and phenolic compound losses were better prevented in fruits treated with 1.5 mM spermidine among different spermidine doses. Gündoğdu et al. (2023) reported that the most dominant phenolic compound detected in plums stored for 20 and 40 days and treated with different spermidine doses was chlorogenic acid, and phenolic compound losses were less in fruits treated with 1.5 mM spermidine among different spermidine doses. In this study and in the above literature studies supporting this study, it is thought that the results obtained in terms of various phenolic compounds may be related to the ‘phenol oxidase enzyme’ which allows internal chemical changes to occur more slowly in spermidine‐treated fruits compared to control fruits (Bal 2012). In addition, it should be taken into account that the amounts of phenolic compounds found in fruits may vary depending on many factors such as fruit type and variety, growing conditions, climatic characteristics and storage conditions (Chen et al. 2021).

Results on organic acids and vitamin C in raspberries were in parallel with the results of some other literature studies on this subject in various fruits. Sayyari et al. (2009) stated that the amount of vitamin C in stored pomegranate fruits in which they applied 2 mM salicylic acid was significantly higher than in control fruits. Orman (2023) reported that the most dominant organic acid among the organic acids detected in nectarine stored for 30 days and treated with different doses of spermidine was malic acid, and the least organic acid losses were reported in fruits treated with 1.5 mM spermidine. Gündoğdu et al. (2023) stated that malic acid was the most dominant organic acid among the organic acids detected in plums stored for 20 and 40 days and treated with different doses of spermidine, and that organic acid losses were prevented more in fruits treated with 1.5 mM spermidine between spermidine applications. Jalali et al. (2023) reported that in strawberries stored for 3 and 6 days and treated with 1.0 and 1.5 mM spermidine doses, vitamin C levels were significantly higher in fruits treated with 1.5 mM spermidine compared to control fruits. Mortazavi et al. (2014) reported that the highest amount of vitamin C in strawberries stored for 10 days and treated with different spermidine doses was observed in fruits treated with 1.5 mM spermidine. Accordingly, in terms of the results determined in this study and in the above literature studies supporting this study, some researchers have reported that the application of polyamines such as spermidine to stored fruits reduces ascorbate oxidase enzyme activity in the fruit and thus vitamin C amounts are better preserved (Zhang et al. 2018). However, the amounts of organic acids and vitamin C in fruits may vary depending on many factors such as fruit type and variety, storage conditions, and genetic characteristics (Zhu et al. 2019).

## 5 Conclusions

This study investigated the effects of spermidine and salicylic acid on the physical and biochemical quality of stored raspberries. The results showed that both treatments effectively reduced quality losses, with the lowest weight loss and fruit decay observed in spermidine + salicylic acid‐treated fruits. Additionally, respiration rates were significantly lower at 5 and 15 days of storage for all treatments. In terms of SSC, the highest levels were found in salicylic acid‐treated fruits after 5 days and in spermidine‐treated fruits after 10 and 15 days. The TA values were significantly lower in spermidine‐treated fruits and higher in salicylic acid‐treated fruits compared to the control. Biochemically, phenolic compounds were better preserved in spermidine + salicylic acid‐treated fruits, while organic acids were better preserved in spermidine and spermidine + salicylic acid‐treated fruits. Vitamin C was better preserved in all treated fruits compared to the control. Overall, spermidine + salicylic acid (1 mM) treatment showed potential as a postharvest tool to enhance raspberry quality and storage life, though results varied with storage periods and specific quality parameters.

## Author Contributions

**Melekşen Akın:** conceptualization (equal), data curation (equal), formal analysis (equal), investigation (equal), methodology (equal), software (equal), visualization (equal), writing – original draft (equal). **Sadiye Peral Eyduran:** formal analysis (equal), investigation (equal), methodology (equal), resources (equal), software (equal), validation (equal), visualization (equal). **Kenan Çelik:** conceptualization (equal), formal analysis (equal), methodology (equal), software (equal). **Akgül Taş:** conceptualization (equal), investigation (equal), methodology (equal), software (equal), validation (equal), visualization (equal), writing – review and editing (equal). **Muttalip Gundogdu:** conceptualization (equal), investigation (equal), methodology (equal), software (equal), validation (equal), visualization (equal).

## Conflicts of Interest

The authors declare no conflicts of interest.

## Data Availability

All data generated or analyzed during this study are included in this published article.
